# Identification of Green-Leaf Volatiles Released from Cabbage Palms (*Sabal palmetto*) Infected with the Lethal Bronzing Phytoplasma

**DOI:** 10.3390/plants12112164

**Published:** 2023-05-30

**Authors:** Jordana A. Ferreira, José A. Ramos, Debora R. C. S. Dutra, Brandon Di Lella, Ericka E. Helmick, Sonia C. N. Queiroz, Brian W. Bahder

**Affiliations:** 1 Laboratory of Residues and Contaminants, Embrapa Environment, Rodovia SP 340, km 127.5, Jaguariúna 13918-110, SP, Brazil; jordanaalvesferreira@gmail.com (J.A.F.); debora.cassoli@embrapa.br (D.R.C.S.D.); sonia.queiroz@embrapa.br (S.C.N.Q.); 2 College of Computing and Engineering, Nova Southeastern University, 3301 College Avenue, Fort Lauderdale, FL 33314-7719, USA; jr1284@nova.edu; 3 Department of Entomology and Nematology, University of Florida–Fort Lauderdale Research and Education Center, 3205 College Ave., Davie, FL 33314-7719, USA; bdilella@ufl.edu (B.D.L.); ehelmick@ufl.edu (E.E.H.)

**Keywords:** stress, LB, plant pathogen, response

## Abstract

Lethal bronzing (LB) is a fatal infection that affects over 20 species of palms (Arecaceae) and is caused by the phytoplasma ‘*Candidatus* Phytoplasma aculeata’. This pathogen causes significant economic losses to landscape and nursery companies in Florida, USA. Recently, the vector was determined to be the planthopper *Haplaxius crudus,* which was more abundant on LB-infected palms. Herein, the volatile chemicals emitted from LB-infected palms were characterized using headspace solid-phase microextraction coupled with gas chromatography-mass spectrometry (HS-SPME/GC-MS). Infected *Sabal palmetto* were identified and confirmed as positive for LB via quantitative PCR. Healthy controls of each species were selected for comparison. All infected palms exhibited elevated levels of hexanal and E-2-hexenal. Threatened palms showed high releasing concentrations of 3-hexenal and Z-3-hexen-1-ol. The volatiles characterized herein are common green-leaf volatiles (GLVs) emitted by plants under stress. This study considers the first documented case of GLVs in palms attributed to phytoplasma infection. Due to the apparent attraction of LB-infected palms to the vector, one or several of the GLVs identified in this study could serve as a lure for the vector and supplement management programs.

## 1. Introduction

Lethal bronzing (LB) is a fatal phytoplasma disease affecting over 20 different species of palm (Arecaceae) [1]. It is widespread in Florida, USA, causing significant economic losses throughout the entire state [2]. Symptoms of LB involve an initial necrosis of the inflorescence and/or premature fruit drop, a browning or bronzing of oldest fronds that rapidly progresses to younger fronds, and the eventual death of the plant, as evidenced by collapse of the spear leaf. Recently, the vector of LB was identified as the planthopper *Haplaxius crudus* (Hemiptera: Cixiidae) [3], which is a very widespread and abundant species in Florida and the Caribbean basin (including Mesoamerica and northern South America) [4]. During survey work in Florida in areas with active disease spread, it was demonstrated that populations of *H. crudus* were higher in areas of LB incidence and appeared to preferentially feed on LB-infected palms over healthy individuals [5]. Furthermore, the difference between LB-infected and healthy trees was demonstrated using a low-cost electronic nose (eNose) [6]. These findings indicate that there is likely one or several volatiles emitted from LB-infected palms that may cause *H. crudus* to orient towards them, thus utilizing this chemical queue.

Plants generally respond to stress through releasing volatile organic compounds (VOCs). Green-leaf volatiles (GLVs) are C6 compounds of alcohols, esters, and aldehydes that attract or repel insects, as well as activate plant defenses, that can possess antimicrobial properties via the hydroperoxide lyase pathway of oxylipin metabolism. As such, GLVs serve as a means of plant-to-plant communication for warning of mechanical damage, herbivory, fungal or bacterial infection, abiotic stress such as drought, heat, and high light [7,8]. The GLVs released can provide precise information about the location of an attacking herbivore, demonstrating that plants can evolve to recognize a pest or microbe (fungi, virus, or bacteria) and initiate their defense mechanisms [9,10]. There are reports that the piercing/sucking behavior of some hemipteran insects showed a lower concentration of GLVs when compared to chewing insects [11,12]. Recently, the vector (*Diaphorina citri*) of ‘*Candidatus Liberibacter* asiaticus’ (causal agent of citrus greening) was shown to prefer non-infected hosts (*Citrus sinensis*), while vectors that were not carrying the pathogen preferred infected citrus [13]. In this study, it was demonstrated that Z-3-hexenyl and methyl salicylate were the principal compounds involved in vector–vector interactions. This suggests that VOCs can influence pathogen−vector interactions, ultimately influencing the epidemiology of a given plant disease. Another study revealed that tobacco (*Nicotiana occidentalis*), apple (*Malus domestica*), and pear (*Pyrus communis*), infected with the phytoplasma ‘*Candidatus phytoplasma* mali’ demonstrated a significant increase in ethyl benzoate concentrations relative to non-infected plants [14]. In addition, for the infected plants, the VOC profile differed from that of the healthy plants and showed high concentrations of unidentified sesquiterpene in tobacco and methyl salicylate in apple trees.

The interaction between GLVs and plant hormones, such as salicylic and jasmonic acids, induces or stimulates plant defenses that are regulated through a complex communication network located between hormonal pathways that influences the plant–pathogen–vector interaction. This system allows plant-to-plant communication about an insect or microbe attack [15,16,17]. A common phenomenon in plants and insects is the ability to convert Z-3-hexenal to its E-2-hexenal isomer after fungal infection or herbivory. However, many questions are yet to be clarified regarding this conversion relation to wound induction or insect derivatives introduced into the wound during feeding. However, the main function and the benefits of this increase in the concentration of E-2-hexenal are yet to be fully elucidated [8]. Despite this, E-2-hexenal appears to play a role in attracting natural enemies of herbivorous insects [18] and providing protection against pathogens [19]. Furthermore, many studies show how E-2-hexenal can be more effective in these roles than Z-3-hexenal [8].

The emission of VOCs and GLVs from damaged, sick, or stressed plants was extensively documented in different plants, such as grape (*Vitis* spp.), tomato (*Solanum* spp.) [20], *Myrcia splendens* [21], *Eugenia uniflora* L. [22] and olive tree [23]. Using gas chromatography (GC-MS) with high sensitivity to and selectivity of volatile compounds has several advantages, including reducing extraction times, boosting high extract purity, and enabling absence or avoidance of the use of organic solvents [24].

**Figure 1:**
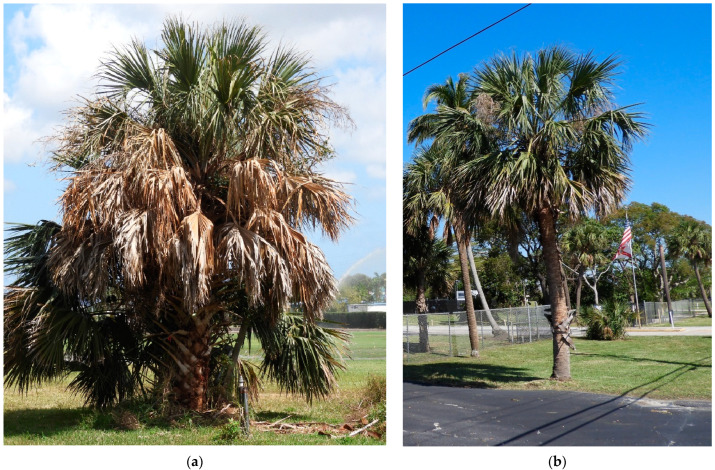
Examples of lethal bronzing-infected (**a**) and healthy (**b**) *Sabal palmetto* specimens.

The potential existence of GLVs being emitted from phytoplasma-infected palms is interesting and, given the apparent attraction of the vector to infected plants, highlights a potential avenue for developing new management practices to reduce vector populations. If one or more volatiles is in fact attractive to *H. crudus*, this volatile could be synthesized and deployed as a lure in palm nurseries and urban habitats to attract adults that would subsequently die in the trap, reducing populations over time. The primary objective of this study is to identify volatiles released from LB-infected *Sabal Palmetto* (Figure 1). The hypothesis is that there is a distinct volatile profile recognizable in LB-infected palms that is not observed in healthy palms.

## 2. Results

### 2.1. qPCR Data and Confirmation of Infection Status

A total of 22 *S. palmetto* were identified as suitable experimental units (Table 1). Two palms were classified as infected (*I*), five palms were classified as non-infected and non-threatened (*NINT*), and 15 palms were classified as non-infected but threatened (*NIT*) (Table 1). The two palms classified as *I* both tested positive using qPCR assays, while all other experimental palms (*NIT* and *NINT*) tested negative (No Ct) using both qPCR assays (Table 1).

### 2.2. Identification of Volatiles Released from S. palmetto Leaves

**Figure 2:**
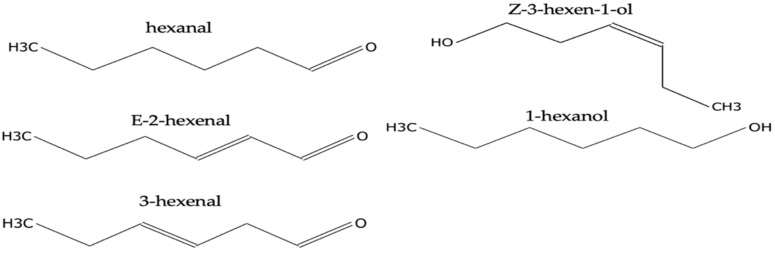
Molecular structures of major green leaf volatiles identified from *Sabal palmetto* leaves.

**Figure 3:**
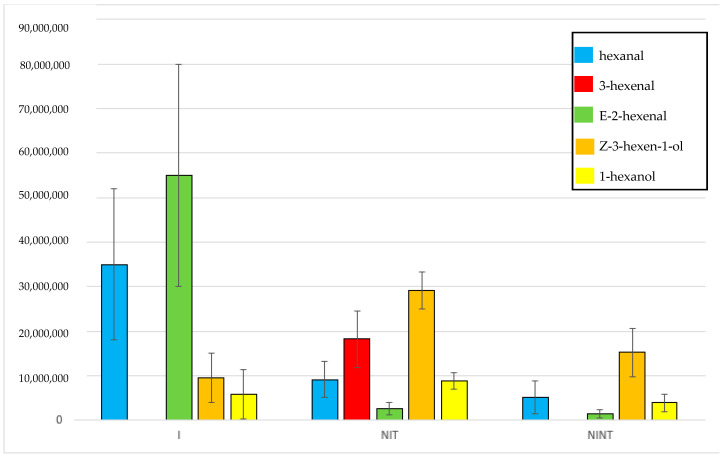
Average volatile concentrations for cabbage palms, infected palms (*I*) (*n* = 2), non-infected and threatened palms (*NIT*) (*n* = 15), and non-infected and non-threatened palms (*NINT*) (*n* = 5).

All volatiles from all samples are listed in Table S1. The five most abundant volatiles identified in this study were hexanal, E-2-hexenal, 3-hexenal, Z-3-hexen-1-ol, and 1-hexanol (Figure 2). Hexanal was present in high concentrations (>1 × 10^7^) in *I* palms (Table S2); however, it was only present substantially lower concentrations in *NIT* (Table S3) and *NINT* (Table S4) palms (Figure 3). Of the 15 palms classified as *NIT*, there was no detection of hexanal in six specimens, while three palms (Spa_7, Spa_8 and Spa_21) displayed concentrations of hexanal comparable to *I* palms (>1 × 10^7^). All other specimens had concentrations of hexanal that were lower that (>1 × 10^7^). The level of observed hexanal among *NIT* and *NINT* palms appeared to be similar. Similar to hexanal, E-2-hexenal was present in substantially higher concentrations in *I* palms (>3 × 10^7^) than in *NIT* and *NINT* palms, which had concentrations below 3 × 10^6^ and 2 × 10^6^, respectively. The concentration of E-2-hexenal did not differ substantially among *NIT* and *NINT* palms. The compound 3-hexenal was detected in high concentrations (>1 × 10^7^) from *NIT* palms but was absent from *I* and *NINT* palms (Figure 3). Concentration of Z-3-hexen-1-ol appeared higher in *NIT* palms than in *I* and *NINT* palms; however, it appeared in similar concentrations in *NINT* and *I* palms (Figure 3). Finally, 1-hexanol was present in similar concentrations in all palms (*I*, *NIT*, and *NINT*) (Figure 3).

Volatile concentrations among samples were consistent in palms in the *I* and *NINT* groups; however, much more variability was observed among samples in the *NIT* group. Palm samples Spa_7, Spa_8, Spa_10, and Spa_21 all displayed significantly higher levels of hexanal that other samples, which were comparable to levels observed in *I* palms; however, these samples tested negative for phytoplasma via qPCR. In addition, hexanal was not detected in Spa_17, which was likely due to error. The same samples also displayed higher levels of E-2-hexenal than other samples, being comparable to concentrations in *I* palms. 2-hexenal was not detected in Spa_7, which was potentially due to error, though if palm was infected, this result could be accurate. The concentrations of Z-3-hexen-1-ol and 1-hexanol in *NIT* were, in contrast, more consistent with levels observed in *I* and *NINT* palms.

**Figure 4:**
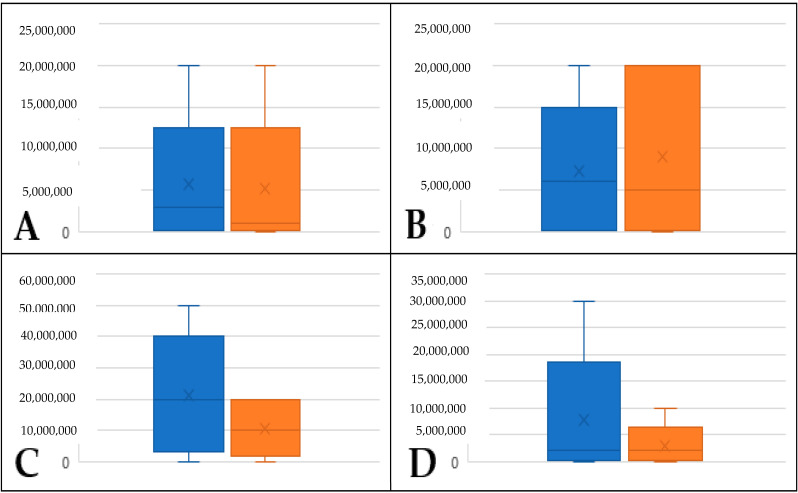
Box chart displaying volatile concentration across replicates in cabbage palms classified as non-infected and non-threatened (*NINT*); (**A**) hexanal, (**B**) E-2-hexenal, (**C**) Z-3-hexen-1-ol, and (**D**) 1-hexanol; blue = replicate 1, orange = replicate 2; *y*-axis is GC/Mass Spec unit concentration.

**Figure 5:**
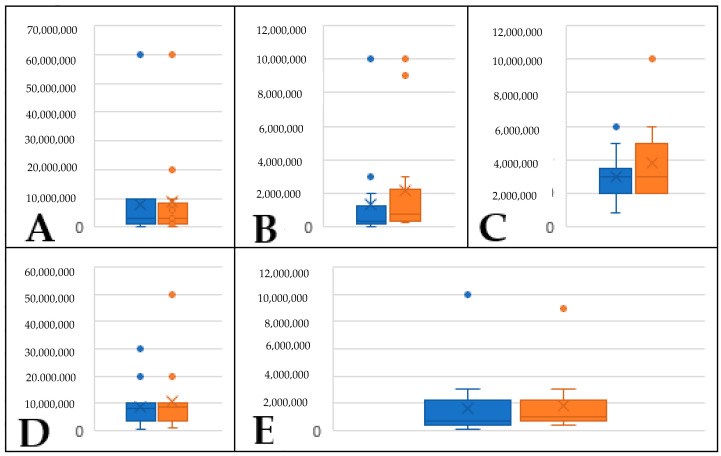
Box chart displaying volatile concentration across replicates in cabbage palms classified as non-infected and threatened (*NIT*); (**A**) hexanal, (**B**) E-2-hexenal, (**C**) Z-3-hexen-1-ol, (**D**) 1-hexanol, and (**E**) 3-hexenal; blue = replicate 1, orange = replicate 2; *y*-axis is GC/Mass Spec unit concentration.

**Figure 6:**
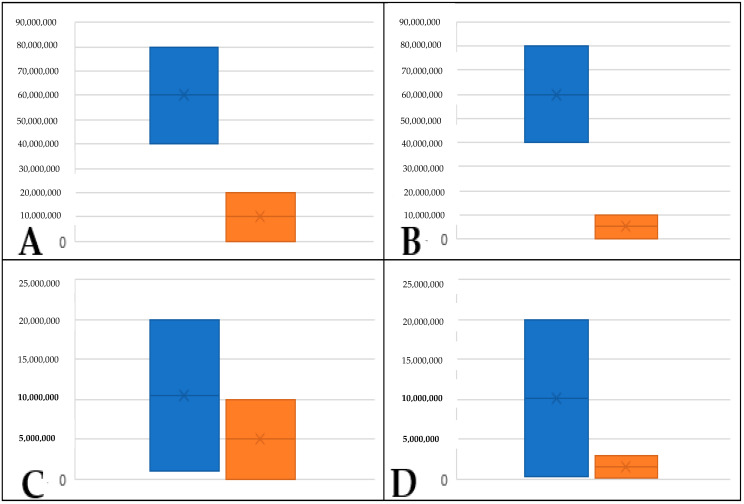
Box chart displaying volatile concentration across replicates in cabbage palms classified as infected (*I*); (**A**) hexanal, (**B**) E-2-hexenal, (**C**) Z-3-hexen-1-o l and (**D**) 1-hexanol; blue = replicate 1, orange = replicate 2; *y*-axis is GC/Mass Spec unit concentration.

When experiments were replicated (approximately one month after the initial experiment), all samples from the *NINT* group of palms had similar concentrations of the five volatiles assessed in this study (Figure 4). Additionally, volatile profiles and concentrations did not differ significantly among replicates for *NIT* palms (Figure 5). For *I* palms, Spa_1 differed significantly in terms of the concentrations of all volatiles except 1-hexanol, with E-2-hexenal and Z-3-hexen-ol becoming undetectable and hexanal dropping from 8 × 10^7^ to 1 × 10^5^ (Figure 6). For Spa_3, volatile profiles and concentrations did not differ significantly among replicates (Figure 6).

### 2.3. Evaluation and Confirmation of GLVs Found in S. palmetto Leaf Samples

Using the deconvolution software to compare the mass spectrum library with the compounds found in the samples as having more than 85% compatibility, approximately 86 compounds were recognized and identified for the infected plants. For the threatened palms, each sample individually presented 82 compounds. The chemical composition profiles of the mixtures of VOCs released in these analyzed samples were different, i.e., only 44% of the compounds between threatened and infected and wounded palms were the same. On the other hand, the compatibility of the release of the same VOCs between threatened and non-infected plants was 33%. When comparing the three possibilities of mixtures of volatiles released in threatened, infected and wounded, and non-infected palm trees, the probability of releasing the same VOCs mixture reduces to 23%.

To confirm the retention times and spectra, the analytical standards of the four major volatiles found in *S. palmetto* leaf samples defined as hexenal, E-2-hexenal, 1-hexanol, and Z-3-hexen-1-ol individually spiked at a concentration of 25 µg g^−1^. Using the deconvolution software, the matches were above 95% compatibility, confirming that the volatiles found in the samples collected in the field corresponded to the analytical standards. Figure S1 shows the analytical standards of some GVLs individually spiked in samples of *S. palmetto* leaves.

## 3. Discussion

The results of this study document the volatiles emitted from phytoplasma infected cabbage palms in Florida, nearby palms that are threatened but uninfected (based on symptoms), and healthy palms far removed from areas with significant disease spread. These data provide the first known classification of GLVs emitted from palms infected with phytoplasmas. Chromatographic profiles involved in the suppression or increase in the Intensity of some major compounds were identified. The overexpression of the results of these analyses showed that the chromatographic profiles of *I*, *NIT*, and *NINT* palms were different in terms of the composition of the GLVs mixture.

Two of the five volatiles assessed, i.e., hexanal and E-2-hexenal, were present in significantly higher concentrations in *I* palms than in *NIT* and *NINT* palms. While these compounds appear be emitted in higher concentrations from LB infected palms, they are emitted by all plants sampled, likely due to the fact that despite other palms not being infected with LB, the study was conducted in a natural/non-sterile setting; therefore, the presence of these volatiles from other palms is likely due to the fact that other types of biotic stresses are present in all plants (general herbivory, non-lethal pathogens) and also cause emission of these volatiles. The significant drop in emission of these volatiles in Spa_1 between replicates is due to the fact that the first experiment was conducted when the palm was still alive, with moderate-to-severe symptoms, while at the time of the second replicate, the plant had succumbed to the infection and, as such, physiological function had ceased. In *NIT* plants, 3-hexenal was present, whereas it was absent in *I* and *NINT* palms, and Z-3-hexen-1-ol appeared to be in higher levels in *NIT* palms compared to *I* and *NINT* palms. Some of the palms (Spa_7, Spa_8, Spa_10, and Spa_21) that were classified as *NIT* due to negative qPCR results at the time of sampling had levels of hexenal and E-2-hexanal that were comparable to *I* palms, and Spa_7 did not have detectable levels of 3-hexenal. These observations could be due to the fact that while these palms were actually infected with LB at the time of volatile collection, either the level of phytoplasma in the palm was not detectable or the infection had not spread to the location of the palm (lower trunk) where samples were taken for qPCR analysis. Volatile emission likely initiates soon after infection and, since the infection starts in the canopy and takes time to become systemic [25], it is possible that volatiles would be indicative of infection status even if qPCR results are negative. Following the termination of the study, Spa_7 subsequently died from LB infection. This finding also is highly valuable from a management perspective because if palms can be classified as infected sooner than is possible with molecular diagnostics, it will allow for more rapid removal of infected palms, possibly before they themselves become infective and allow further spread, greatly improving management of disease’s spread.

The GLVs found in this work have some documented biological functions, such as aroma and flavor [26] and pathogen defense [27,28,29] for hexanal; herbivore defense [30,31], plants priming [32], pathogen defense [33], and aroma and flavor [29] for E-2-hexenal; Herbivore defense [34,35], pathogen defense [19,36] for Z-3-hexen-1-ol; aroma and flavor [37] and pathogen defense [38] for 1-hexanol; aversive or toxic for arthropod predators for 3-hexenal and E-4-oxohex-2-nal [39]; and pathogen defense [40] for E,E-2,4 hexadienal. At all stages of host–pathogen interaction, the plant responds by releasing patterns of GLVs [34]. Some of these compounds, hexanal, i.e., 1-hexanol, E-2-hexenal, and Z-3-hexen-1-ol, play important roles in plant defense against microbial proliferation in injured areas [29]. Hexanal and E-2-hexenal demonstrated antimicrobial activity against species such as *Listeria monocytogenes*, *Staphylococcus aureus*, *Salmonella enteritidis*, and *Escherichia coli* [40]. It also showed that an increase in E-2-hexenal can benefit plants through means of attracting natural enemies of the pest insect species, as well as signaling to gravid females about egg-laying sites [8]. In Wei and Kang (2011) [36], Z-3-hexen-1-ol demonstrated uses in plant–plant communication, including avoiding herbivore attacks. Furthermore, it proved to be the most important information signal for inducing gene expression in threatened plants. In addition, this compound showed positive indirect defense and priming effects. Although it is difficult to conclude whether Z-3-hexen-1-ol is an attractant or a repellent, accumulating evidence suggests that Z-3-hexen-1-ol is at least in part an important volatile that can modulate the behavior of herbivorous insects, and their release must be the plants’ defensive responses.

Overall, the documented GLVs found in this study appear to be consistent with roles that the corresponding volatiles have in the above-mentioned studies, such as direct pathogen defense, modification of insect behavior (either a repellant of the pest or attractant of beneficial insects), or a combination of both factors. The role that the GLVs documented in this study have on the LB phytoplasma itself, as well as their effect on the vector, *H. crudus*, the efficacy of other cabbage palms to resist infections by generating defenses, and, ultimately, the epidemiology of LB, is unknown. However, these data are highly valuable because they lay foundations for exploring a wide variety of management options, including the following; (1) assessing compounds as injectable treatments for early infections to cure plants; (2) stimulating plants to generate their own defenses; (3) developing feeding deterrents for *H. crudus*; and (4) developing attractants for natural enemies of *H. crudus*. Future research efforts will seek to adapt these data to an applied context in an attempt to improve IPM practices for control of LB in palms in Florida.

## 4. Materials and Methods

### 4.1. Sample Selection and Verification of Infection Status

Palms selected for this study were assigned to one of three different categories: infected (*I*), non-infected and threatened (*NIT*), or non-infected and non-threatened (*NINT*). Palms displaying symptoms of LB and testing positive (*I)* at FLREC were identified at and sampled using the protocol outlined by Bahder and Helmick [41]. Palms immediately adjacent to *I* palms that appeared healthy (*NIT*) were identified and sampled in the same way as *I* palms. Finally, palms that appeared healthy and were considered not to be threatened due to being located outside of the area affected by disease (*NINT*) were identified and sampled in the same way as *I* and *NIT* palms. Total DNA was extracted from palm trunk tissue using the protocol outlined by Soto et al. [42]. To confirm whether palms were infected with the LB phytoplasma or healthy, all palms were screened via quantitative PCR (qPCR) analysis and high-resolution melt curve analysis (HRMA) using the parameters outlined in Bahder et al. [41]. Due to the nature of the disease and the fact that the experiment was conducted under natural settings, the sample size for each group (treatment) was limited based on two factors: (1) the number of palms for each group that were present at the time of the study, and (2) the number of palms in each group that were accessible (i.e., short enough to reach the canopy and collect leaf tissue).

### 4.2. Identification of Volatile Profiles via HS-SPME/GC-MS

#### 4.2.1. Reagents and Materials

For the determination of the retention times of the main volatile compounds, the standards of Z-3-hexen-1-ol (≥98%), 1-hexanol (≥99%), hexanal (≥97%), E-2-hexenal (≥95%) were purchased from Sigma-Aldrich (St. Louis, MO, USA). For the HS-SPME procedure, amber glass vial of 20 mL, a screw thread cap with microcenter PTFE/silicone septa and SPME manual injection kit were obtained from Restek Corporation (Bellefonte, PA, USA). For the HS-SPME procedure, an amber glass vial of 20 mL and screw thread cap with microcenter PTFE/silicone septa and SPME manual injection kit were obtained from Restek Corporation (Bellefonte, PA, USA), as were SPME fibers coated with 65 um Divinylbenzene/Polydimethylsiloxane (DVB/PDMS). A heater from Fisher Scientific and a balance from Sartorius also were used.

#### 4.2.2. Sample Preparation

Leaves from *S. palmetto* specimens from Section 4.1 were collected in sterile plastic bags in sequence, taken to the laboratory, and cut into small pieces using garden shears. The leaves were cut to a size of approximately 5 mm inside sterile bags to avoid contamination, and the greenest leaves were selected for this study, with leaves closest to the spear leaf prioritized when collecting the spear leaf was difficult. Samples were collected from plants of different ages and heights. Analyses were performed on fresh leaves on the same day. If necessary, leaf samples were stored in the ultra-freezer at −80 °C. Each sample was weighed 4 g in a headspace vial of 20 mL with a screw thread cap and microcenter PTFE/silicone septa obtained from Restek Corporation (Bellefonte, PA, USA). For initial analysis, leaves of different palms at different stages after qPCR assays were collected, such as (a) no bacteria; (b) with bacteria; and (c) in the advanced stage of infection (i.e., palm almost dead). The garden shears were disinfected with 70% ethyl alcohol after each cut of the leaves that represented a plant.

#### 4.2.3. HS-SPME Procedure

Prior to the isolation of volatiles, 4 g of leaves were weighed in weight boats and transferred to 20 mL vials with the help of a clamp and kept in sealed vials until reaching room temperature for frozen samples. The SPME fibers was exposed to the headspace for 10 min at 60 °C. The extracted analytes were immediately desorbed, separated, and detected using GC-MS.

#### 4.2.4. GC-MS Analysis

All analyses were performed through gas chromatography coupled with mass spectrometry (GC 7890B, and MS 5977A, Agilent Technologies, Waldbronn, Germany). Chromatographic separation was performed using an Agilent J&W HP-5MS Ultra Inert column with the following dimensions: 30 m × 250 µm × 0.25 µm, non-polar column, (5%-phenyl)-methylpolysiloxane)). Desorption was performed directly in the gas chromatograph injector port over 10 min at 250 °C using the splitless mode. Some combinations of GC-MS created oven temperature programming were tested to improve the separation of chromatographic peaks, as previously proposed by Terra et al. (2020) [43], as shown in Table 2.

The detection was performed using 70 eV of ionization energy in a mass range of 40–550 a.m.u. The ion source temperature 280 °C and the interface temperature was 250 °C. Helium was used as a carrier gas at a flow rate of 1.0 mL/min. After GC-MS analysis, the chromatograms were deconvolved using Agilent Masshunter Qualitative and identified through comparison of mass spectra library with the National Institute of Standards and Technology (NIST 14) database used for identification. Subsequently, the major compounds were evaluated using analytical standards with >95% purity, confirming the retention time and spectrum of the compounds. Background correction was accomplished via injecting exposed fibers into the vial without sample contact and comparing results with experimental units to verify the volatiles that were plant derived, as shown in Supplementary Table S1.

## 5. Conclusions

In this study, GLVs were identified from *Sabal palmetto* that were infected with the lethal bronzing phytoplasma, nearby palms that were healthy but at risk of infection, and healthy palms outside of the disease area. The GLVs with the highest release were 1-hexanol, 3-hexenal, hexanal, E-2-hexenal, and Z-3-hexen-1-ol. Infected palms generated higher levels of hexanal and 2-hexenal than non-infected palms and palms that were at risk of infection generated 3-hexenal, whereas infected plants and healthy plants outside of the disease area did not have this compound.

## Figures and Tables

**Table 1:** Specimens of *Sabal palmetto* selected for analysis, as well as their infection statuses and quantitative PCR results.

		qPCR Results			
Palm Sample	Infection Status	TaqMan Ct	HRMA Ct	HRMA Tm (°C)	Result
Spa_1	*I*	24.33	22.43	77.6	+
Spa_2	*NINT*	No Ct	No Ct	64.5	-
Spa_3	*I*	33.97	30.21	77.6	+
Spa_4	*NIT*	No Ct	No Ct	82.8	-
Spa_5	*NIT*	No Ct	No Ct	82.8	-
Spa_6	*NINT*	No Ct	No Ct	82.8	-
Spa_7	*NIT*	No Ct	No Ct	82.7	-
Spa_8	*NIT*	No Ct	No Ct	82.9	-
Spa_9	*NINT*	No Ct	No Ct	82.7	-
Spa_10	*NIT*	No Ct	No Ct	82.9	-
Spa_11	*NIT*	No Ct	No Ct	82.8	-
Spa_12	*NIT*	No Ct	No Ct	82.8	-
Spa_13	*NIT*	No Ct	No Ct	82.8	-
Spa_14	*NIT*	No Ct	No Ct	82.7	-
Spa_15	*NINT*	No Ct	No Ct	82.9	-
Spa_16	*NINT*	No Ct	No Ct	83.0	-
Spa_17	*NIT*	No Ct	No Ct	82.8	-
Spa_18	*NIT*	No Ct	No Ct	82.7	-
Spa_19	*NIT*	No Ct	No Ct	82.7	-
Spa_20	*NIT*	No Ct	No Ct	82.7	-
Spa_21	*NINT*	No Ct	No Ct	82.8	-
Spa_22	*NIT*	No Ct	No Ct	82.9	-
(+) control	N/A	23.44	21.75	77.6	+
(-) control	N/A	No Ct	No Ct	55.34	-

**Table 2:** GC-MS oven temperature programming.

	Rate (°C/min)	T (°C)	Hold Time (min)	Run Time (min)
Initial		40	2	2
Ramp 1	5	150	1	25
Ramp 2	10	250	5	40

## Data Availability

Not applicable.

## Supplementary Materials

The following supporting information can be downloaded at: https://www.mdpi.com/article/10.3390/plants12112164/s1. Figure S1: GC-MS chromatograms of elevated GLVs in *S. palmetto* leaf samples; Table S1: Volatiles released from *S. palmetto* leaves; Table S2: Green leaf volatile average (±SE) concentrations (GC/MassSpec Counts) from infected (*I*) palms; Table S3: GLVs concentrations of cabbage palms in close proximity (*NIT*) to lethal bronzing-infected palms; Table S4: GLVs concentrations of non-infected and non-threatened (*NINT*) cabbage palms.
