# Supplementary material for: Identification of Green-Leaf Volatiles Released from Cabbage Palms (Sabal palmetto) Infected with the Lethal Bronzing Phytoplasma
Source: Plants (Basel). 2023 May 30;12(11):2164. doi: 10.3390/plants12112164 (PMC10255706; doi:10.3390/plants12112164)
Supplement: Supplementary file 1 [file plants-12-02164-s001.zip › Table S1.pdf]

Supplementary Table S1. Volatiles released from *S. palmetto* leaves

Threatened		Infected		No Bacteria/threat	
Retention times	*Compounds*	*Retention times*	*Compounds*	*Retention times*	*Compounds*
3.1228	Furan, 2-ethyl-	3.123	Furan, 2-ethyl-	3.2775	Acetoin
3.2412	2-Pentenal, (E)-	3.2466	Acetoin	3.3115	2,4-Dimethylfuran
3.2729	Acetoin	3.5319	2-Pentenal, 2-methyl-	3.6827	1-Butanol, 3-methyl-
3.5313	2-Butenal, 2-ethyl-	3.6851	1-Butanol, 3-methyl-	3.7498	Butyl isocyanatoacetate
3.6876	1-Butanol, 3-methyl-	3.8657	2-Pentenal, (E)-	4.2768	Toluene
3.8686	2-Pentenal, (E)-	3.9605	(S)-5-Hydroxymethyl-2[5H]-furanone	4.3137	1-Pentanol
3.9671	(S)-5-Hydroxymethyl-2[5H]-furanone	4.0426	2-Pentenal, (E)-	4.391	2-Penten-1-ol, (Z)-
4.0523	2-Pentenal, (E)-	4.2819	Toluene	4.9969	Furan, 2-methoxy-
4.3038	Cyclobutanone, 2,2,3-trimethyl-	4.3056	Cyclobutanone, 2,2,3-trimethyl-	5.0145	Hexanal
4.3745	2-Penten-1-ol, (Z)-	4.3739	2-Penten-1-ol, (Z)-	5.9146	Difluoroisocyanatophosphine
4.7933	1-Octene	4.7971	2-Octene	6.1736	2-Pentanone, 4-hydroxy-4-methyl-
5.0006	3-Hexenal	5.0475	Hexanal	6.4102	2-Hexenal, (E)-
5.5675	Butanoic acid, 3-methyl-, 3-methyl-3-butenyl ester	5.7106	Formic acid, pentyl ester	6.4819	3-Hexen-1-ol, (Z)-
5.7056	Formic acid, pentyl ester	5.9327	Hexane, 3,3,4-trimethyl-	6.5975	Ethylbenzene
6.1045	3,3-Diethoxy-1-propyne	6.2027	2-Hexenal, (E)-	6.6687	Heptane, 2,4-dimethyl-
6.1869	2-Hexenal, (E)-	6.453	2-Hexenal, (E)-	6.8144	p-Xylene
6.3765	2-Hexenal, (E)-	6.523	3-Hexen-1-ol, (Z)-	6.8525	1-Hexanol
6.5673	3-Hexen-1-ol, (Z)-	6.877	1-Hexanol	7.4889	Benzene, 1,3-dimethyl-
6.8178	Benzene, 1,3-dimethyl-	7.4911	p-Xylene	7.7069	Nonane
6.8859	1-Hexanol	7.7645	Heptanal	7.7754	Hexanal, 3-methyl-
7.7631	Heptanal	7.8673	Oxime-, methoxy-phenyl-	7.8681	Oxime-, methoxy-phenyl-_
7.8468	Oxime-, methoxy-phenyl-_	7.9938	2,4-Hexadienal, (E,E)-	8.2146	Anisole
8.0057	2,4-Hexadienal, (E,E)-	8.1211	Butanoic acid, 4-hydroxy-	9.5399	Octane, 2,2,6-trimethyl-
8.3374	3-Hexen-1-ol, formate, (Z)-	8.3431	Ethyl (Z)-hex-3-enyl carbonate	10.0588	Pentanoic acid
8.5703	Formic acid, hexyl ester	8.5758	Formic acid, hexyl ester	10.3055	Hexanethioic acid, S-methyl ester

8.7055	(1R)-2,6,6-Trimethylbicyclo[3.1.1]hept-2-ene	8.7103	(1R)-2,6,6-Trimethylbicyclo[3.1.1]hept-2-ene	10.3953	5-Hepten-2-one, 6-methyl-
9.0643	Furan, 2-butyltetrahydro-	9.4311	2-Heptenal, (E)-	10.5425	Mesitylene
9.288	Oxalic acid, cyclohexyl butyl ester	9.5186	Benzaldehyde	10.762	Decane
9.5133	Benzaldehyde	9.533	(E)-4-Oxohex-2-enal	10.8718	Octanal
9.523	(E)-4-Oxohex-2-enal	9.8618	2-Octene	10.9417	3-Heptene, 2,2,4,6,6-pentamethyl-
9.8515	2-Octene	10.1414	1-Octen-3-ol	11.3185	4-Cyanocyclohexene
10.0141	Bicyclo[3.1.1]heptane, 6,6-dimethyl-2-methylene-, (1S)-	10.2047	Pentanoic acid	11.5029	Pentane, 2,2,4-trimethyl-
10.1288	1-Octen-3-ol	10.3003	n-Caproic acid vinyl ester	11.6378	D-Limonene
10.3821	5-Hepten-2-one, 6-methyl-	10.3756	3-Octanone	11.755	3-Heptene, 2,2,4,6,6-pentamethyl-
10.4697	2-Hexenal, (E)-	10.5125	Furan, 2-pentyl-	12.1075	Benzeneacetaldehyde
10.754	Decane	10.6665	2,4-Heptadienal, (E,E)-	12.4909	Disulfide, bis(1,1,3,3-tetramethylbutyl)
10.86	Octanal	10.7602	Octane, 2,5-dimethyl-	12.7184	Sulfurous acid, 2-ethylhexyl hexyl ester
11.0007	3-Hexen-1-ol, acetate, (Z)-	10.8686	Octanal	12.8001	Ethanone, 2-(formyloxy)-1-phenyl-
11.2959	Cyclohexen-1-carbonitrile	11.0977	Furan, 2-propyl-	13.3896	Benzene, 1-ethenyl-4-ethyl-
11.6279	D-Limonene	11.7959	1,2-Propanediol, 1-phenyl-	13.6712	Heptane, 4-methyl-
11.7837	1,2-Propanediol, 1-phenyl-	11.8779	(E)-4-Oxohex-2-enal	13.8192	Undecane
11.8645	(E)-4-Oxohex-2-enal	12.1036	Benzeneacetaldehyde	13.8977	Octane, 5-ethyl-2-methyl-
12.4373	2(3H)-Furanone, 5-ethyldihydro-	12.5804	2-Pentene, 1-ethoxy-4-methyl-, (Z)-	13.9656	Nonanal
12.566	2H-Pyran-2-methanol, tetrahydro-	12.7125	Hexane, 2,2,5,5-tetramethyl-	14.2565	Naphthalene, 1,2-dihydro-
12.7828	Acetophenone	12.8011	Benzoic acid, (4-benzoyloxy-2-chlorophenyl) ester	14.629	Undecane, 3,7-dimethyl-
12.9382	Formic acid, octyl ester	12.8487	Bicyclo[4.2.0]octa-1,3,5-trien-7-ol	15.5217	2-Oxo-4-phenyl-6-(4-chlorophenyl)-1,2-dihydropyrimidine
13.37	Benzene, 1-ethenyl-4-ethyl-	13.3793	Benzene, 1-ethenyl-3-ethyl-	15.5912	Undecane, 4-methyl-

13.5956	Benzene, 1-ethenyl-4-ethyl-	13.6032	Benzene, 1-ethenyl-4-ethyl-	15.7043	Benzaldehyde, 3-ethyl-
13.9537	Nonanal	13.9641	Nonanal	15.7166	Dodecane, 5-methyl-
14.7821	Benzene, 1,1'-(1,5-hexadiene-1,6-diyl)bis-	14.6292	Undecane, 4,7-dimethyl-	15.9091	Undecane, 3-methyl-
15.4713	Undecane, 5-methyl-	15.3789	Benzene, 1-ethenyl-4-methoxy-	16.1446	Benzaldehyde, 4-ethyl-
15.6916	Benzaldehyde, 3-ethyl-	15.4131	2,6-Nonadienal, (E,Z)-	16.2447	Cinnamaldehyde, (E)-
15.7065	Nonane, 5-(2-methylpropyl)-	15.5931	Undecane, 4-methyl-	16.27	Naphthalene
16.1323	Benzaldehyde, 4-ethyl-	15.699	Benzaldehyde, 4-ethyl-	16.7339	Cinnamaldehyde, (E)-
16.2322	Cinnamaldehyde, (E)-	15.7159	Nonane, 5-butyl-	16.7642	Dodecane
16.2759	1-(1-Methoxypropan-2-yloxy)propan-2-yl acetate	15.9081	Undecane, 3-methyl-	16.9409	Decanal
16.3846	Butanoic acid, 3-hexenyl ester, (Z)-	16.1403	Benzaldehyde, 4-ethyl-	17.1608	Undecane, 3,6-dimethyl-
16.6186	Methyl salicylate	16.2399	Cinnamaldehyde, (E)-	17.3237	Ethanol, 2-phenoxy-
16.7212	Cinnamaldehyde, (E)-	16.5256	Cyclopentane, 1,2,3-trimethyl-	17.5565	Isophthalaldehyde
16.7549	Dodecane	16.6264	Methyl salicylate	17.7661	Isophthalaldehyde
16.9327	Decanal	16.7289	Cinnamaldehyde, (E)-	18.103	1H-Indene-4-carboxaldehyde, 2,3-dihydro-
17.5468	1,4-Benzenedicarboxaldehyde	16.761	Dodecane	18.5719	m-Ethylacetophenone
17.7586	Isophthalaldehyde	16.9393	Decanal	18.7005	1H-Indene, 1-ethyl-2,3-dihydro-
18.087	1H-Indene-4-carboxaldehyde, 2,3-dihydro-	17.5551	Isophthalaldehyde	18.8086	Undecane, 6,6-dimethyl-
18.5626	m-Ethylacetophenone	17.7646	Isophthalaldehyde	19.1466	3-Buten-2-one, 4-phenyl-
18.6907	1H-Indene, 1-ethyl-2,3-dihydro-	17.9454	Cyclopentane, butyl-	19.3273	1-Undecanol
19.1387	3-Buten-2-one, 4-phenyl-	18.0975	1H-Indene-4-carboxaldehyde, 2,3-dihydro-	19.6201	p-Isopropenylphenol
19.321	1-Decanol	18.5718	Ethanone, 1-(4-ethylphenyl)-	19.9636	Phthalic anhydride
20.5051	Ethanone, 1-(2,4-dimethylphenyl)-	18.6989	1H-Indene, 1-ethyl-2,3-dihydro-	20.691	Heptylcyclohexane

20.6847	Heptylcyclohexane	19.0876	Ethanone, 1-(4-ethylphenyl)-	20.9786	Sulfurous acid, cyclohexylmethyl isobutyl ester
20.9733	Sulfurous acid, cyclohexylmethyl heptadecyl ester	19.146	3-Buten-2-one, 4-phenyl-	21.1253	4'-Methylpropiophenone
21.1199	4'-Methylpropiophenone	19.3249	1-Undecanol	21.364	4-Benzyloxybenzonitrile
21.5379	Propanoic acid, 2-methyl-, 3-hydroxy-2,2,4-trimethylpentyl ester	19.6191	p-Isopropenylphenol	22.302	Nonane, 5-methyl-5-propyl-
22.1902	Hexane, 2,4,4-trimethyl-	19.9653	1,2-Benzenedicarboxylic acid	23.9667	Decane, 1-chloro-
23.5815	4-Methylene-3,4-dihydroisocoumarin	20.5141	Ethanone, 1-(3,4-dimethylphenyl)-	25.0178	1-Heptadecyne
23.6134	Dimethyl phthalate	20.9781	Sulfurous acid, cyclohexylmethyl isobutyl ester	27.3208	Pentadecanal-
27.074	2,2,4-Trimethyl-1,3-pentanediol diisobutyrate	21.1292	4'-Methylpropiophenone	28.4333	Dodecane, 1-chloro-
27.3168	1,14-Tetradecanediol	23.0617	1(3H)-Isobenzofuranone, 3,3-dimethyl-	29.6692	Tetradecanoic acid
28.8034	Carbonic acid, nonyl prop-1-en-2-yl ester	27.3203	Z-2-Dodecenol	30.97	Pentadecanoic acid
29.0432	Pentadecanal-	28.4356	1-Chloroundecane	31.1572	Phthalic acid, isobutyl 4-octyl ester
31.1526	1,2-Benzenedicarboxylic acid, bis(2-methylpropyl) ester	29.0476	Pentadecanal-	32.1338	n-Hexadecanoic acid
31.4159	Homosalate	29.6638	Tetradecanoic acid	32.2359	Phthalic acid, butyl cyclobutyl ester
32.1223	n-Hexadecanoic acid	31.1571	Phthalic acid, cyclohexyl isohexyl ester	34.4183	Phenol, 4,4'-(1-methylethylidene)bis-
		32.1258	n-Hexadecanoic acid		
		32.2363	Phthalic acid, butyl cyclobutyl ester		
		34.4187	Phenol, 4,4'-(1-methylethylidene)bis-		
