# Supplementary material for: Identification of Green-Leaf Volatiles Released from Cabbage Palms (Sabal palmetto) Infected with the Lethal Bronzing Phytoplasma
Source: Plants (Basel). 2023 May 30;12(11):2164. doi: 10.3390/plants12112164 (PMC10255706; doi:10.3390/plants12112164)
Supplement: Supplementary file 1 [file plants-12-02164-s001.zip › Table S2.pdf]

Supplementary Table S2. Green leaf volatile average (±SE) concentrations (GC/MassSpec Counts) from infected (*I*) palms.

Palm ID	Hexanal	2-Hexenal	cis-3-Hexen-1-ol	1-Hexanol
Spa_1	8E+7 (±0.0000000)	8E+7 (±0.0000000)	1E+6 (±0.000000)	1E+5 (±0.00000)
Spa_3	4E+7 (±0.0000000)	4E+7 (±0.0000000)	2E+7 (±0.0000000)	2E+7 (±0.0000000)
