# Supplementary material for: Identification of Green-Leaf Volatiles Released from Cabbage Palms (Sabal palmetto) Infected with the Lethal Bronzing Phytoplasma
Source: Plants (Basel). 2023 May 30;12(11):2164. doi: 10.3390/plants12112164 (PMC10255706; doi:10.3390/plants12112164)
Supplement: Supplementary file 1 [file plants-12-02164-s001.zip › Table S3.pdf]

Supplementary Table S3. GLVs concentrations of cabbages palms in close proximity (*NIT*) to lethal bronzing infected palms.

Palm ID	Hexanal	E-2-hexenal	3-hexenal	Z-3-hexenol	1-hexanol
Spa_4	1E+6 (±0.000000)	2E+5 (±0.00000)	4E+6 (±0.000000)	2E+7 (±0.0000000)	4E+6 (±0.000000)
Spa_5	3E+6 (±0.000000)	4E+5 (±0.00000)	9E+6 (±0.000000)	3E+7 (±0.0000000)	7E+6 (±0.000000)
Spa_7	3E+7 (±0.0000000)	2E+7 (±0.0000000)	No Detection	2E+7 (±0.0000000)	1E+7 (±0.0000000)
Spa_8	1E+7 (±0.0000000)	2E+6 (±0.000000)	2E+7 (±0.0000000)	3E+7 (±0.0000000)	1E+7 (±0.0000000)
Spa_10	6E+7 (±0.0000000)	1E+7 (±0.0000000)	1E+8 (±0.00000000)	3E+7 (±0.0000000)	8E+6 (±0.000000)
Spa_11	9E+6 (±0.000000)	5E+6 (±0.000000)	3E+7 (±0.0000000)	2E+7 (±0.0000000)	8E+6 (±0.000000)
Spa_12	3E+6 (±0.000000)	2E+4 (±0.0000)	9E+5 (±0.00000)	8E+6 (±0.000000)	2E+6 (±0.000000)
Spa_13	7E+5 (±0.00000)	6E+4 (±0.0000)	5E+6 (±0.000000)	1E+7 (±0.0000000)	2E+6 (±0.000000)
Spa_14	1E+6 (±0.000000)	2E+5 (±0.00000)	1E+7 (±0.0000000)	2E+7 (±0.0000000)	4E+6 (±0.000000)
Spa_17	No Detection	1E+6 (±0.000000)	3E+7 (±0.0000000)	6E+7 (±0.0000000)	2E+7 (±0.0000000)
Spa_18	6E+6 (±0.000000)	3E+5 (±0.00000)	1E+7 (±0.0000000)	2E+7 (±0.0000000)	7E+5 (±0.00000)
Spa_19	2E+6 (±0.000000)	9E+4 (±0.0000)	2E+7 (±0.0000009)	3E+7 (±0.0000000)	1E+7 (±0.0000000)
Spa_20	3E+6 (±0.000000)	3E+5 (±0.00000)	7E+6 (±0.000000)	3E+7 (±0.0000000)	8E+6 (±0.000000)
Spa_21	1E+7 (±0.0000000)	1E+6 (±0.000000)	2E+7 (±0.0000000)	6E+7 (±0.0000000)	3E+7 (±0.0000000)
Spa_22	4E+4 (±0.0000)	4E+5 (±0.00000)	8E+6 (±0.000000)	5E+7 (±0.0000000)	9E+6 (±0.000000)
