# Supplementary material for: Identification of Green-Leaf Volatiles Released from Cabbage Palms (Sabal palmetto) Infected with the Lethal Bronzing Phytoplasma
Source: Plants (Basel). 2023 May 30;12(11):2164. doi: 10.3390/plants12112164 (PMC10255706; doi:10.3390/plants12112164)
Supplement: Supplementary file 1 [file plants-12-02164-s001.zip › Table S4.pdf]

Supplementary Table S4. GLVs concentrations of non-infected and non-threatened (*NINT*) cabbage palms.

Replicates ID	3-hexenal	hexenal	2-E-hexenal	Z-3-hexenol	1-hexanol
Spa_2	No Detection	1E+6 (±0.000000)	8E+03 (±0.000)	7E+4 (±0.0000)	3E+4 (±0.0000)
Spa_6	No Detection	3E+6 (±0.000000)	6E+5 (±0.00000)	3E+7 (±0.0000000)	7E+6 (±0.000000)
Spa_9	No Detection	2E+6 (±0.000000)	5E+6 (±0.000000)	2E+7 (±0.0000000)	2E+6 (±0.000000)
Spa_15	No Detection	2E+7 (±0.0000000)	2E+6 (±0.000000)	5E+7 (±0.0000000)	1E+7 (±0.0000000)
Spa_16	No Detection	6E+4 (±0.0000)	1E+4 (±0.0000)	6E+6 (±0.000000)	6E+5 (±0.00000)
