# Supplementary figures and images for: Identification of Green-Leaf Volatiles Released from Cabbage Palms (Sabal palmetto) Infected with the Lethal Bronzing Phytoplasma
Source: Plants (Basel). 2023 May 30;12(11):2164. doi: 10.3390/plants12112164 (PMC10255706; doi:10.3390/plants12112164)

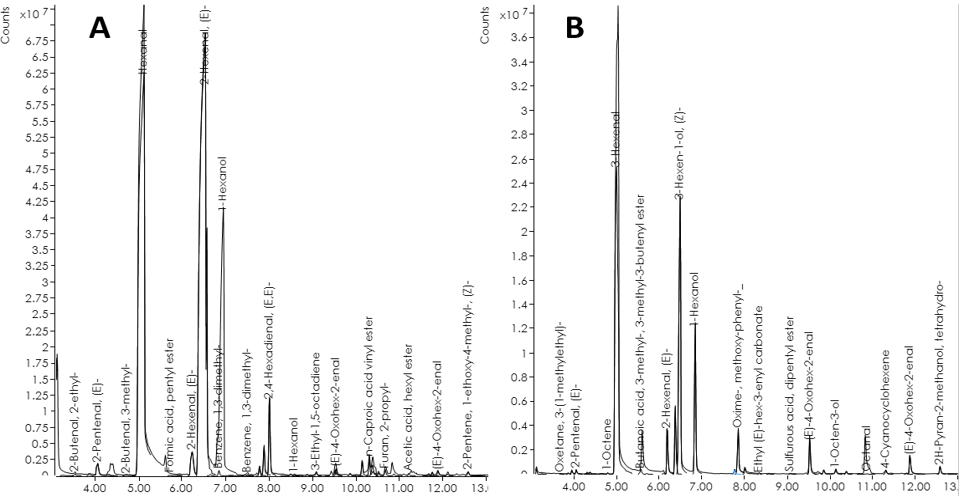

Supplement: Supplementary file 1 [file plants-12-02164-s001.zip › Figure S1.tif]
